# One Health and surveillance of zoonotic tuberculosis in selected low-income, middle-income and high-income countries: A systematic review

**DOI:** 10.1371/journal.pntd.0010428

**Published:** 2022-06-06

**Authors:** Rodrigo de Macedo Couto, Giulia Osório Santana, Otavio T. Ranzani, Eliseu Alves Waldman

**Affiliations:** 1 Department of Epidemiology, Universidade de São Paulo, São Paulo, Brazil; 2 Barcelona Institute for Global Health, ISGlobal, Barcelona, Spain; 3 Pulmonary Division, Heart Institute (InCor), Hospital das Clínicas HCFMUSP, Faculdade de Medicina, Universidade de São Paulo, São Paulo, Brazil; Oregon State University College of Veterinary Medicine, UNITED STATES

## Abstract

**Background:**

Little is known about zoonotic tuberculosis (zTB) due to *Mycobacterium bovis* burden across the globe. The aim of this study was to describe zTB surveillance programs in selected WHO signatory countries and to assess the relationship of the disease with the country’s income level and the risk of *M*. *bovis* transmission.

**Methods:**

We searched the main articles databases and grey literature for guide documents published between 1980 and 2019. For inclusion, the articles and guide documents had to be in English, French, Portuguese, Spanish, or Italian. Only original articles and narrative and systematic reviews were accepted and the guide documents were required to be available on official websites. We excluded articles that did not focus on epidemiology, control and surveillance. We used bovine TB cases in livestock and wildlife populations as a proxy for the country’s risk of zTB using data from the World Organization for Animal Health (OIE) published from 2015 to 2018. Countries were classified according to income level (World Bank’s classification) and strength of zTB surveillance. The study was registered in PROSPERO under number CRD42018090603.

**Findings:**

We included 13 articles and 208 guide documents including data from 119/194 countries (61.3%). We found a lack of surveillance data about zTB in over half (89.9%) of the 119 WHO signatory countries. Most surveillance systems perform passive surveillance and are not integrated into the One Health perspective, which was operating in 4/119 (3.4%) countries, all high-income. Many of these countries (71/119, 59.7%) have M. bovis circulating in their cattle herds, but only ~10% of them have implemented zTB surveillance activities.

**Interpretation:**

Our findings highlight weaknesses in zTB surveillance worldwide, with a consequent lack of information that could support an adequate understanding of disease burden, especially in countries at major risk for *M*. *bovis* transmission. To meet this challenge, efforts will be needed to promote intersectoral policies, implementing the One Health strategy.

## Author summary

Little is known, across the world, about of tuberculosis in humans caused by *Mycobacterium bovis*, ie, zoonotic tuberculosis (zTB). Insufficient data are a major challenge for developing strategies for the control of zTB, in particular in low-income and middle-income countries. Surveillance data are essential to develop effective TB control strategies, quantifying disease burden, and identifying risk factors and vulnerable groups. Strengthening the One Health approach is essential for making zoonotic disease control strategies highly effective by promoting concerted actions across human and animal health programs and integrated surveillance. A systematic review was conducted to describe existing zTB surveillance and the degree of its coordination with animal TB surveillance in countries of low-income, middle- and high-income, accounting for the country risk of *M*. *bovis* transmission. We found that zTB surveillance is highly conditioned by country’s income level, and identified a discrepancy between the level of risk of animal-human TB transmission and the extent of surveillance. In addition, less than 5% of the countries assessed have implemented integrated human and animal TB control programs, which suggests low adoption of the One Health approach across the world. Without adequate surveillance for zTB, across the world, it will be unlikely to guarantee diagnosis and treatment for all TB patients.

## Introduction

Zoonotic diseases have gained importance due to their impact on human health and the economy, with their inclusion in the global public health agenda becoming a priority. The successful control of zoonotic diseases depends to a large extent on the implementation of long-term intersectoral public policies, which act synergistically by promoting social and economic development and a better quality of life and health of the population [1]. To this end, it is also crucial to strengthen research and innovation for more effective health care services and surveillance systems and to help countries around the world to progress towards all *Sustainable Development Goals (SDG)* [2]. Strengthening the One Health approach is essential in order to implement highly effective zoonotic disease control strategies by promoting concerted actions across human and animal health programs and integrated surveillance systems [3].

The World Health Organization (WHO) defines zoonotic tuberculosis (zTB) as a form of TB in humans that is predominantly caused by *Mycobacterium bovis*, which belongs to the *M*. *tuberculosis* complex [4]. Recently, *Mycobacterium orygis*, a new member of the complex described in 2012, has been associated with zTB especially in Asia. However, robust estimates of its prevalence in humans and animals are lacking [5].

Cattle are the most important reservoir of *M*. *bovis* but other animal species are also involved in disease transmission, a fact that renders it a highly complex process [6]. According to 2019 estimates, there were nearly 140,000 new cases and 11,400 deaths due to zTB worldwide; however, these numbers are expected to be underestimated because of the lack of zTB data [7]. This data gap arises from weak or inexistent organized surveillance systems focused on zTB [6]. Historically, zTB has been associated with the extrapulmonary form in children, usually caused by the consumption of unpasteurized milk from infected cows. However, with the widespread use of milk pasteurization across much of the globe over the course of the 20th century, its prevalence has declined drastically. Currently, molecular techniques suggest the importance of airborne transmission between humans and animals, until recently little discussed. The reduction of *M*. *bovis* infection in cattle should be the pillar of disease prevention in humans and should be achieved by the inspection of slaughterhouses and the application of the tuberculin test to animals [6].

Insufficient data and knowledge are a major challenge for developing strategies for the control of zTB, in particular in low-income and middle-income countries where people are more vulnerable due to a greater risk of TB [8]. Surveillance data are essential to develop effective TB control strategies, to quantify disease burden, to identify risk factors and vulnerable groups, and to monitor morbidity and mortality trends. Effective zTB surveillance is a major step for countries to move towards the WHO’s End TB Strategy [9,10].

To the best of our knowledge, there are no published reports based on global data to support approaches to the strengthening of zTB surveillance and control in different countries. In an attempt to fill this knowledge gap, we conducted a systematic review aimed at describing the existing zTB surveillance systems and the availability of data, including their characteristics, basic components and degree of coordination with animal TB surveillance in countries of low, middle and high income, accounting for the country’s risk of *M*. *bovis* transmission.

## Materials and methods

### Search strategy and selection criteria

We followed the Preferred Reporting Items for Systematic Reviews and Meta-Analyses (PRISMA) guidelines (http://prisma-statement.org/PRISMAStatement/Checklist.aspx) (S1 PRISMA Checklist). This systematic review was registered in the International Prospective *Register* of Systematic Reviews (*PROSPERO*) (protocol number CRD42018090603) (https://www.crd.york.ac.uk/prospero/)).

We included scientific articles and guide documents from national public health bodies published in English, French, Portuguese, Spanish, or Italian with a focus on topics of zTB epidemiology, control and surveillance. For scientific articles we considered only original articles or narrative or systematic reviews. For guide documents, we considered only if available on official websites.

We excluded articles focusing on: i) infections and/or diseases associated with mycobacteria other than *M*. *bovis*; ii) animal TB infection; iii) bacterial genetics, phylogeny, and genotyping; iv) pathogen isolation and detection, diagnosis, pathogenesis, or disease transmission; v) interdisciplinary research on other zoonotic diseases; vi) immunology, vaccination, treatment, and drug resistance, and vii) clinical case presentations.

We searched PubMed, Embase and Index Medicus for the South-East Asian region, Web of Science, African Index Medicus, Index Medicus for the Eastern Mediterranean region, and SciELO and Lilacs for Latin America, comprising the period from 1980 to 2019. We considered a broader period in the search for scientific articles in an attempt to find countries that have traditionally published data on zTB, thus creating a timeline of continuity of surveillance services for these countries.

Since we only considered *M*. *bovis* as a zTB agent, our search terms were “*Mycobacterium bovis*,” “surveillance,” “control” and “epidemiology,” adjusted for each database (S1 Data). We also searched the grey literature for guide documents and technical reports available on the WHO website and Ministry of Health websites (or equivalents) for all WHO signatory countries. These searches were performed in 2018 and 2019 and the last guide available were selected. The shorter search period for the grey literature reflected our objective of demonstrating the structuring of surveillance services in different countries at the current date.

Two researchers (RMC and GOS) evaluated and selected the articles. They independently read the titles and abstracts of the articles retrieved and then reviewed the full text of the selected articles for relevance, methodological rigor and inclusion/exclusion criteria. Disagreements were resolved by a third researcher (EAW). We conducted additional searches to locate the references cited in the articles retrieved and other publications were selected based on our inclusion and exclusion criteria.

We used the Strengthening the Reporting of Observational Studies in Epidemiology (STROBE) checklist (https://www.equator-network.org/) to assess the study design of the articles and potential biases. This checklist did not apply to guide documents.

We searched for potentially relevant technical guides prepared by national TB control programs (NTPs) and/or surveillance programs on Ministry of Health websites from 194 WHO signatory countries. All available documents were included in the study.

### Data analysis

Two researchers (RMC and GOS) independently reviewed the data extracted from all documents and articles, namely: country, study design (for articles only), study population (for articles only), exposures associated with human transmission of *M*. *bovis*, basic surveillance components (data source, goals, passive and active surveillance, definition of suspected cases, definition of confirmed cases, laboratory tests for case confirmation, surveillance data analysis, interpretation, dissemination, and use in research) [9,11], and collaboration approaches between animal and human health sectors including integrated human and animal health laboratories. In addition, we extracted from guide documents relevant zTB surveillance information, including the identification of *M*. *bovis* as a potential causative agent, description of transmission modes, reservoir characterization, identification of risk groups, and description of clinical forms.

We adopted the World Bank classification of 2018 for the categorization of countries by income level (databank.worldbank.org/data/download/site-content/CLASS.xls), which divides groups according to gross national income per capita, calculated using the World Bank Atlas method. The groups are: low income, $1,035 or less; lower middle income, $1,036–4,045; upper middle income, $4,046–12,535; high income, $12,536 or more. We considered cases of TB in livestock and wildlife populations as a proxy for the country’s risk of zTB; these data were obtained from the World Organization for Animal Health (OIE) database for the period from January 2015 to December 2018 (http://www.oie.int/wahis_2/public/wahid.php/Diseaseinformation/statusdetail)). We grouped each country according to income classification and strength of zTB surveillance.

All data collected were tabulated and summarized. Because of the small number of studies selected, we were not able to conduct a meta-analysis. We used Qgis (version 3.8) to generate thematic maps according to the country’s income level and risk of zTB and the R software (version 3.6.1) for creating graphs.

## Results

Thirteen articles were selected from the data sources searched for this study (S1 PRISMA Flow Diagram) according to the inclusion and exclusion criteria, including data from nine countries. The risk of bias of the articles selected was low (S1 Table).

**Fig 1:**
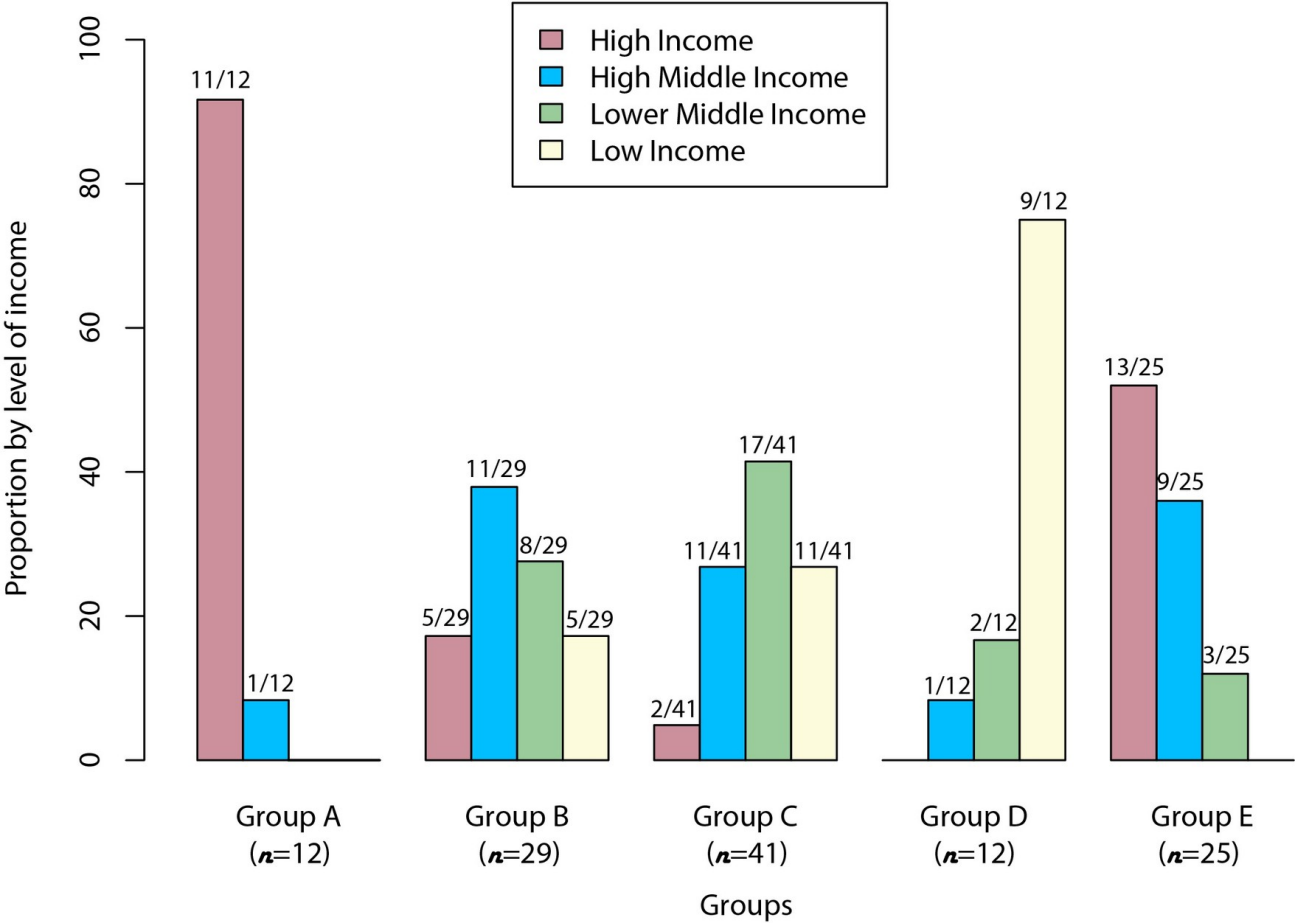
Distribution of countries classified in Groups A, B, C, D and E by income level. Group A (n = 12): countries with tuberculosis surveillance with specific zoonotic tuberculosis surveillance activities (three components of surveillance: established sources for data collection; routine analysis of information available; and wide dissemination of the analyzed data). Group B (n = 29): countries with tuberculosis surveillance without zoonotic tuberculosis surveillance activities (identification of *Mycobacterium bovis* as a causative agent of tuberculosis in their working documents and in the national tuberculosis control programs). Group C (n = 41): countries with tuberculosis surveillance but no information on zoonotic tuberculosis from working documents available. Group D (n = 12): countries with no clear reference to existing tuberculosis surveillance or national tuberculosis control programs; no reference to zoonotic tuberculosis. Group E (n = 25): countries similar to Group D with no clear reference to existing tuberculosis surveillance or national tuberculosis control programs, and no reference to zoonotic tuberculosis, but showing some characteristics that might affect zTB epidemiology (i.e., island, city-state, population and small area).

Application of the language exclusion criterion resulted in the exclusion of 75/194 (38.7%) WHO signatory countries, 5/75 (7%) low income; 21/75 (28%) lower middle income; 22/75 (29%) upper middle income; and 27/75 (36%) high income. Thus, we searched for documents on the websites of the respective Ministries of Health from 119/194 (61.3%) countries, whose income distribution is presented in Fig 1. We identified 208 guide documents from 82/119 (68.9%) countries (minimum of 1 and maximum of 8 per country). Tables 1 and 2 show relevant zTB surveillance data from the 82 countries. We included at least one guide document from each of the nine countries with articles selected for the study, but we did not find any article or guide document available for the remaining 37/119 (31.1%) countries (S2 PRISMA Flow Diagram).

**Table 1:** Basic components of surveillance of human tuberculosis caused by *Mycobacterium bovis* in 82 countries assessed, by income level.

Components of surveillance systems		Number of countries by income level and percentage: n (%)					
			Low income * n = 16	Lower-Middle Income * n = 25	Upper-Middle Income * n = 23	High Income * n = 18	Total n = 82
Define the sources of information?		Yes	0 (0%)	0 (0%)	1 (4%)	11 (61%)	12 (15%)
		No	16 (100%)	25 (100%)	22 (96%)	7 (39%)	70 (85%)
Are the objectives of the surveillance system described?		Yes	0 (0%)	0 (0%)	0 (0%)	0 (0%)	0 (0%)
		No	16 (100%)	25 (100%)	23 (100%)	18 (100%)	82 (100%)
Types of surveillance system	Passive surveillance	Yes	0 (0%)	0 (0%)	1 (4%)	11 (61%)	12 (15%)
		No	16 (10%)	25 (100%)	22 (96%)	7 (39%)	70 (85%)
	Active surveillance	Yes	0 (0%)	0 (0%)	0 (0%)	2 (11%)	2 (2%)
		No	16 (100%)	25 (100%)	23 (100%)	16 (89%)	80 (98%)
Case definitions	Is there a suspected case definition?	Yes	0 (0%)	0 (0%)	0 (0%)	0 (0%)	0 (0%)
		No	16 (100%)	25 (100%)	23 (100%)	18 (100%)	82 (100%)
	Is there a confirmed case definition?	Yes	0 (0%)	0 (0%)	2 (9%)	10 (55%)	12 (15%)
		No	16 (100%)	25 (100%)	21 (91%)	8 (45%)	70 (85%)
Diagnostic laboratory techniques	Does it specify the culture medium for the growth of *M*. *bovis*?	Yes	0 (0%)	1 (4%)	4 (17%)	9 (50%)	14 (17%)
		No	16 (100%)	24 (96%)	19 (83%)	9 (50%)	68 (83%)
	Does it perform zTB diagnosis in routine activities of the National Tuberculosis Control Program?	Yes	0 (0%)	0 (0%)	1 (4%)	11 (61%)	12 (15%)
		No	16 (100%)	25 (100%)	22 (96%)	7 (39%)	70 (85%)
	Does it use genotyping techniques?	Yes	0 (0%)	0 (0%)	1 (4%)	9 (50%)	10 (12%)
		No	16 (100%)	25 (100%)	22 (96%)	9 (50%)	72 (88%)
Does it regularly review surveillance data and release it periodically?		Yes	0 (0·0%)	0 (0%)	1 (4%)	11 (61%)	12 (15%)
		No	16 (100%)	25 (100%)	22 (96%)	7 (39%)	70 (85%)
Are surveillance data used to support control strategies?		Yes	0 (0%)	0 (0%)	1 (4%)	5 (28%)	6 (7%)
		No	16 (100%)	25 (100%)	22 (96%)	13 (72%)	76 (93%)
Are surveillance data used to support research?		Yes	0 (0%)	0 (0%)	2 (9%)	9 (50%)	11 (13%)
		No	16 (100%)	25 (100%)	21 (91%)	9 (50%)	71 (87%)
Is there mention of articulation of zTB surveillance with animal health sectors?		Yes	0 (0%)	0 (0%)	0 (0%)	4 (22%)	4 (5%)
		No	16 (100%)	25 (100%)	23 (100%)	14 (78%)	78 (95%)

* Income classification according to the World Bank.

** Source: articles and technical texts included in the study.

**Table 2:** Key aspects of surveillance of human tuberculosis caused by *Mycobacterium bovis* in 82 countries assessed, by income level.

Relevant information about the organization of surveillance systems for human tuberculosis by *Mycobacterium bovis*		Number of countries by income level and percentage: n (%)				
		Low income* n = 16	Lower-Middle Income* n = 25	Upper-Middle Income* n = 23	High Income* n = 18	Total n = 82
Is *Mycobacterium bovis* mentioned as a possible etiologic agent?	Yes	5 (31%)	8 (32%)	13 (56%)	16 (89%)	42 (51%)
	No	11 (69%)	17 (68%)	10 (44%)	2 (11%)	40 (49%)
Does it describe transmission mechanisms?	Yes	2 (12%)	4 (16%)	7 (30%)	10 (56%)	23 (28%)
	No	14 (88%)	21 (84%)	16 (70%)	8 (44%)	59 (72%)
Does it specify reservoirs?	Yes	0 (0%)	0 (0%)	7 (30%)	8 (44%)	15 (18%)
	No	16 (100%)	25 (100%)	16 (70%)	10 (56%)	67 (82%)
Does it specify risk groups?	Yes	1 (6%)	0 (0%)	1 (4%)	9 (50%)	11 (13%)
	No	15 (94%)	25 (100%)	22 (96%)	9 (50%)	71 (87%)
Does it describe characteristic clinical forms?	Yes	0 (0%)	0 (0%)	4 (17%)	6 (33%)	10 (12%)
	No	16 (100%)	25 (100%)	19 (83%)	12 (67%)	72 (88%)

* Income classification according to the World Bank.

** Source: articles and technical texts included in the study.

### Assessment of zoonotic tuberculosis surveillance data

Based on information collected from the articles and guide documents included in the study, we reached a consensus and divided the countries into five groups (Fig 1 and S2 Table) as follows: i) group A: 12/119 (10.1%) countries with TB surveillance and specific zTB surveillance activities; ii) group B: 29/119 (24.4%) countries with TB surveillance without zTB surveillance activities, but relevant zTB information available; iii) group C: 41/119 (34.5%) countries with TB surveillance but no information on zTB; iv) group D: 12/119 (10.1%) countries with no articles or guide documents available in the data sources, and v) group E: 25/119 (21.0%) countries with no articles or guide documents available in the data sources, similar to group D, but showing specific characteristics that could influence zTB epidemiology (i.e., small islands, city-state, small population, and/or small area). In countries of groups D and E, we did not find documents or articles that could infer whether they have TB or zTB surveillance, because during the search, we did not access any data.

Surveillance activities carried out in countries from group A involved three basic components [12]: i) established sources for data collection; ii) routine analysis of available information, and iii) wide dissemination of the analysed data. Assessment of surveillance data from these countries permitted us: i) to identify rising zTB rates in the United Kingdom and Mexico [12–14]; ii) to detect a predominance of zTB cases among migrants from neighbouring countries in the United States [15]; iii) to characterize latent infection reactivation among zTB cases in the United Kingdom [12,14] and Italy [16], and iv) to detect higher mortality rates among zTB cases when compared to TB cases associated with *M*. *tuberculosis* in the Netherlands [17].

Surveillance of zTB was passive in countries from group A. Only 2/12 countries (17%), both high income, reported routine active human case-finding from confirmed animal TB cases [18,19]. The data analysed from these 12 countries made no reference to either specific goals set for zTB surveillance (only for TB associated with *M*. *tuberculosis*) or the definition of suspected cases of zTB (only reporting that cases were confirmed by culture isolation of *M*. *bovis*). Ten out of 12 countries (83%), all of which were high income, reported using genotyping as an important adjunct diagnostic test to identify the source of infection [20].

Surveillance can be considered effective in these countries since it provides scientific data informing about disease control strategies including: i) indication of targeted tuberculin testing in people exposed to animals with respiratory infection or aerosol-generating procedures with the potential for airborne transmission of infection (Canada) [18]; ii) indication of diagnostic tests for zTB for individuals eating *undercooked meat* or consuming *unpasteurized dairy products* from infected animals (Canada, Spain, and Ireland) [18,21,22], and iii) mandatory reporting of zTB resulting from occupational exposure and mandatory use of personal protective equipment to prevent exposure to *M*. *bovis* (United Kingdom) [19]. In addition, surveillance data were used as a source of information for scientific research (Australia, France, United States, New Zealand, United Kingdom, The Netherlands, Mexico, Italy, and Spain) [13,15–17,19,23–30].

Coordination between animal and human health sectors incorporating core concepts of the One Health approach was evidenced in 4/12 countries (33%), all of which were high income. The key aspects included: i) laboratory-based surveillance focused on cross-transmission between humans and animals with genotyping of all human and animal isolates (The Netherlands) [31]; ii) cross-reporting of health issues among animal and human health care services in Canada [18], including infections in domesticated non-bovine animals (dogs, cats and goats), and wild animals in captivity in the United Kingdom [12,32]; iii) alerts issued for signs and symptoms in individuals exposed to sick animals (Ireland) [22], and iv) collaboration between veterinarians and human health providers to investigate potential animal sources of infection in environments where there is the potential for human exposure (United Kingdom) [12]. No reference was made to the use of pyrazinamide resistance as a marker of suspected zTB cases.

*Mycobacterium bovis* is described as a possible causal agent of TB cases in guide documents from 3/29 (10%) countries in group B (Burkina Faso, Tanzania, and Sri Lanka) [33–35]. There were also descriptions of clinical-epidemiological aspects and diagnosis of zTB: i) modes of transmission, in particular through the digestive tract (Cameroon) [36]; ii) implications of different clinical forms such as ganglionic (Brazil) [37] and gastrointestinal TB (South Africa) [38]; iii) specific risk groups requiring targeted surveillance such as children consuming raw milk (Uganda) [39] and higher risk of occupational exposure among slaughterhouse employees and rural workers (Brazil) [37], and iv) use of a particular culture medium for *M*. *bovis* isolation–Lowenstein-Jensen medium with sodium pyruvate (India) [40].

The data analysed for countries in groups C, D and E made no reference to zTB surveillance in routine TB surveillance activities of the NTPs, either because there was no mention in the guide documents (group C) or because there were no guide documents available in the sources searched (groups D and E). It is noteworthy that countries in group D were either low income or middle income (Niger, Democratic Republic of the Congo, and Central African Republic). On the other hand, countries in group E were medium-to-high income islands or small territories with low population density (Andorra, Bahamas, Luxembourg, Monaco, and Marshall Islands) (S2 Table).

### Assessment animal tuberculosis surveillance data

**Fig 2:**
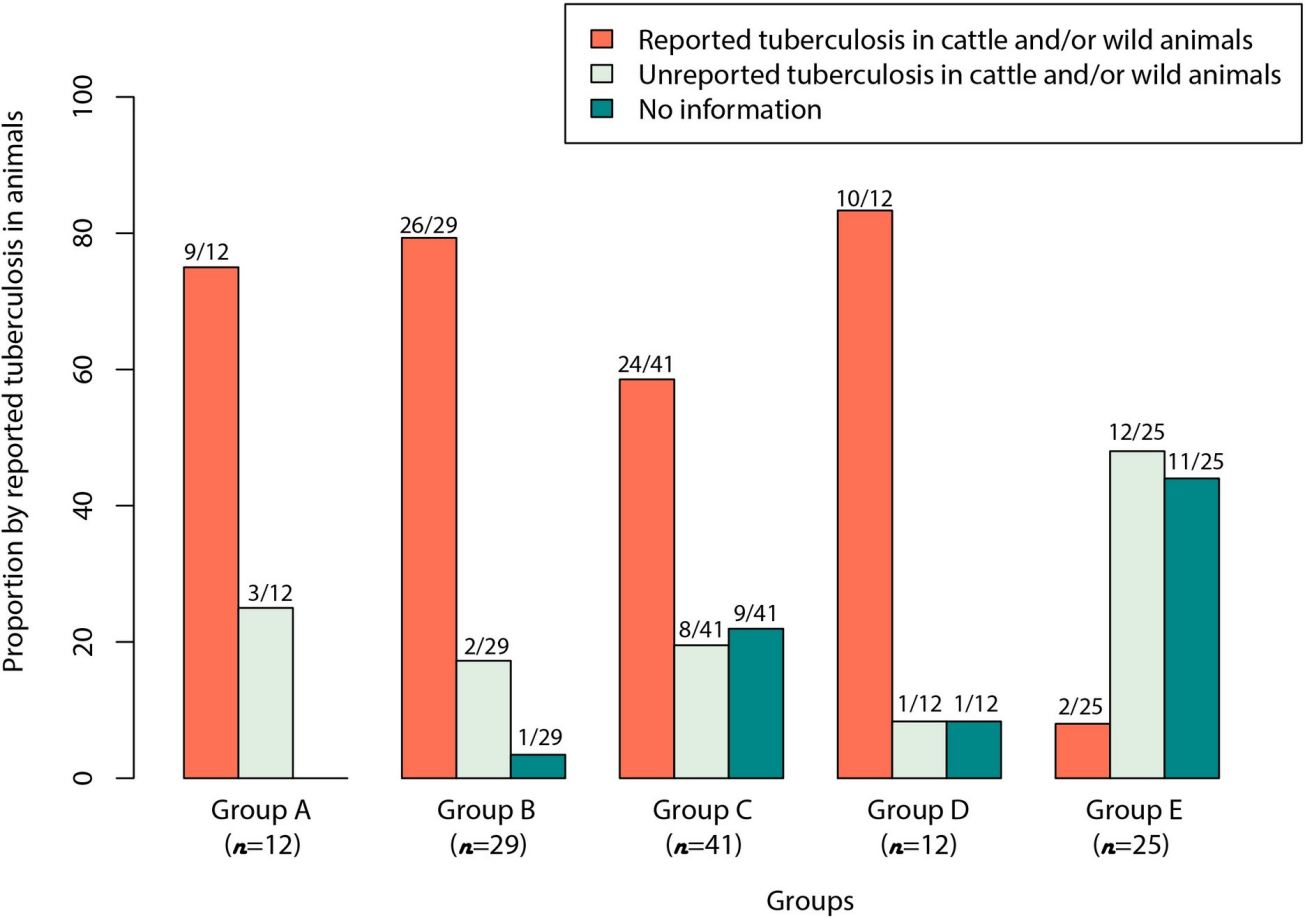
Distribution of countries classified in Groups A, B, C, D and E by reported tuberculosis in animals. Group A (n = 12): countries with tuberculosis surveillance with specific zoonotic tuberculosis surveillance activities (three components of surveillance: established sources for data collection; routine analysis of information available; and wide dissemination of the analyzed data). Group B (n = 29): countries with tuberculosis surveillance without zoonotic tuberculosis surveillance activities (identification of *Mycobacterium bovis* as a causative agent of tuberculosis in their working documents and in the national tuberculosis control programs). Group C (n = 41): countries with tuberculosis surveillance but no information on zoonotic tuberculosis from working documents available. Group D (n = 12): countries with no clear reference to existing tuberculosis surveillance or national tuberculosis control programs; no reference to zoonotic tuberculosis. Group E (n = 25): countries similar to Group D with no clear reference to existing tuberculosis surveillance or national tuberculosis control programs, and no reference to zoonotic tuberculosis, but showing some characteristics that might affect zTB epidemiology (i.e., island, city-state, population and small area).

**Fig 3:**
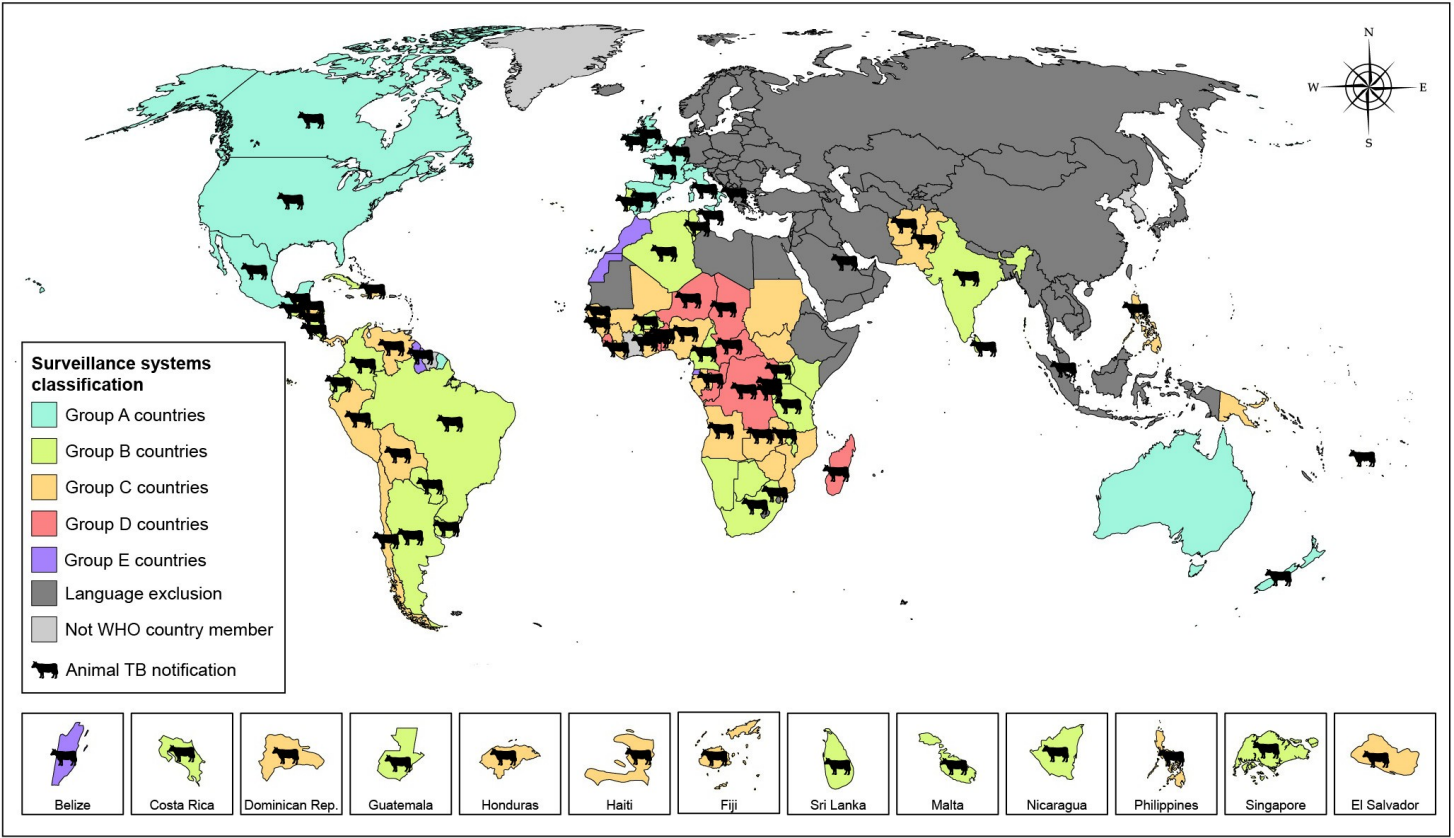
Global map illustration of the surveillance data classification of human tuberculosis caused by *Mycobacterium bovis* and reported animal tuberculosis. Group A (n = 12): countries with tuberculosis surveillance with specific zoonotic tuberculosis surveillance activities (three components of surveillance: established sources for data collection; routine analysis of information available; and wide dissemination of the analyzed data). Group B (n = 29): countries with tuberculosis surveillance without zoonotic tuberculosis surveillance activities (identification of *Mycobacterium bovis* as a causative agent of tuberculosis in their working documents and in the national tuberculosis control programs). Group C (n = 41): countries with tuberculosis surveillance but no information on zoonotic tuberculosis from working documents available. Group D (n = 12): countries with no clear reference to existing tuberculosis surveillance or national tuberculosis control programs; no reference to zoonotic tuberculosis. Group E (n = 25): countries similar to Group D with no clear reference to existing tuberculosis surveillance or national tuberculosis control programs, and no reference to zoonotic tuberculosis, but showing some characteristics that might affect zTB epidemiology (i.e., island, city-state, population and small area). Note: due to the small territorial extension and to facilitate identification, some countries were highlighted in the tiles below. http://www.naturalearthdata.com/about/terms-of-use/.

For the period from 2015 to 2018 (OIE data, 2015–2018), there were reports of TB in livestock and/or wildlife populations in 9/12 countries in group A (75%), 26/29 in group B (90%), 24/41 in group C (58%), 10/12 in group D (83%), and 2/25 in group E (8%), for a total of 71/119 (59.7%) (Figs 2 and 3).

## Discussion

This is possibly the first study focusing on zTB surveillance accessing data from a good part of the world’s countries. There is a lack of surveillance data about zTB in over half (89.9%) of the 119 WHO signatory countries accessed. Additionally, most surveillance systems perform passive surveillance and are not integrated into the One Health perspective.

Many of these countries (71/119, 59.7%) have *M*. *bovis* circulating in their cattle herds, but only ~10% of them have implemented zTB surveillance activities. On the other hand, ~25% of these countries point out the importance of *M*. *bovis* for TB control in their guide documents, recognizing how zTB surveillance is essential for controlling TB in humans, yet, they are mostly high-income or middle-income countries. It is important to point that, unlike human tuberculosis, TB in livestock is not a notifiable/controlled disease in many low- and middle-income countries and therefore there is often a lack of data on TB in the livestock population in these countries, underestimating the dimension of the disease. Therefore, the zTB risk may be underestimated in our analysis and the probability of underestimation is likely higher for countries that have large numbers of cattle herds.

It is accepted that effective zTB surveillance and control rely to a large extent on concerted actions based on the One Health approach in human and animal populations [4,6]. Yet, integrated animal and human surveillance is in place in a very small proportion of the 119 countries assessed (<5%). Nevertheless, these few countries could be seen as a model for the others. Governments must, as a first step, design clear objectives, structure health systems, strengthen the regulatory area, and structure surveillance guides. The second step is to integrate the two surveillance systems, animal and human surveillance. Although experiences in high-income countries are important to formulate policies for low- and middle-income countries, it is essential to adapt them to the structure and degree of complexity of human and animal health services in these countries. The observation that few countries have adopted the One Health approach is a matter of concern considering the animal origin of many diseases [1,3].

We identified data gaps even in the few countries with zTB surveillance, such as lack of a clear definition of suspected cases, which would be a valuable tool to improve surveillance sensitivity [9,11], and lack of the use of pyrazinamide resistance information as an indicator of suspected zTB infection. This finding underscores the importance of coordinating animal surveillance with human m*ultidrug*-*resistant TB* surveillance [41].

In countries that have implemented a specific surveillance component for *M*. *bovis* in their national TB programs, recent *M*. *bovis* infections continue to occur even after the introduction of control measures for the sanitary control of herds (including the compulsory testing of dairy cattle), or for the sanitary control of food products (including milk pasteurization), indicating that these animals are likely to have acquired infection from other sources or other transmission routes. Thus, trends and risk factors should continue to be monitored [19,26,29]. Another factor that contributed to the beginning of this surveillance, especially integrated with animal surveillance, is the spillover of TB from wild animals to cattle herds, reintroducing the disease in already controlled areas and posing a risk to human health [42]. Buying animals may carry a considerable risk too. The introduction of an undetected long-incubation disease like TB may affect up to 26% of farms if infection remains undetected for 3 years [43].

Since our results indicate that zTB surveillance is carried out predominantly in high-income countries, it is possible that this disease is being neglected in many low- and middle-income countries. Some important risk factors associated with disease transmission occur especially in these countries, where the disease is more prevalent. In many African countries, milk pasteurization is not used regularly and 80%–90% of the volume produced is sold by small dairy farms and pastoral communities [6].

We must acknowledge some limitations in interpreting the findings of our study. Nearly one-third (38%) of WHO signatory countries were not assessed in this study, with a large proportion (64%) being low-income and middle-income countries. More studies including central, eastern Europe, Russia and middle east countries are necessary, in order to access zTB surveillance data. It is important to mention that specially in these countries, despite previously falling TB incidence, there was an upsurge of TB cases and deaths throughout the region after the economic recession and other crises [44]. Nevertheless, for the countries included, the review provides relevant information for the development of public health policies. Another limitation is that we did not assess risk factors associated with zTB occurrence (e.g., organization and functioning of veterinary services, cultural practices, and sanitary control of animal products). Finally, we only considered the current WHO definition of zTB. Recent publications have highlighted the role of other mycobacteria in the transmission of TB between humans and animals, such as *M*. *orygis* [5], which was not considered here as a search term in the databases. We believe, however, that the proposed surveillance should be aware of the evolution of knowledge and be able to integrate new definitions (flexibility of surveillance systems) [9,11] such as the incorporation of *M*. *orygis* as a potential zoonotic TB agent and others that may yet be identified.

Our findings provided quantitative data supporting the literature claim that significant *gaps exist* in surveillance *data*. New diagnostic technologies are urgently needed *to differentiate M*. *bovis from M*. *tuberculosis disease*, *improving routine diagnosis of M*. *bovis in human cases of TB*, *making possible quantify zTB burden*. *Similarly*, *surveillance systems need improve zTB case definitions*, *incorporating suspected case definition*. *These limitations* show that zTB will be an additional challenge along the path to reach the goal of ending TB in 2030 if no urgent action is taken [4]. Finding and treating every case of TB, whether caused by *M*. *tuberculosis* or *M*. *bovis*, will count towards the achievement of this ambitious goal [1,2,4,10].

zTB continues to negatively affect both the health and economy of a considerable number of people, and the health and welfare of animals. Since the risk of zTB occurrence is intrinsically related to the occurrence of cases in domestic animals, and that the occurrence in these animals is related to the spread of the disease from wild reservoirs, identifying wild hosts and mapping the occurrence in these animals is fundamental for the control of the transmission of *M*. *bovis*. Countries must intensify their efforts to incorporate the One Health approach by integrating surveillance in humans, animals and environmental areas. To make the SDG Agenda a reality, broad ownership must translate into a strong engagement by all stakeholders.

## Supporting information

S1 PRISMA Checklist PRISMA 2009 checklist. (DOCX)

S1 Data Search terms and databases. (DOCX)

S1 PRISMA Flow Diagram PRISMA diagram for the articles. (DOCX)

S2 PRISMA Flow Diagram PRISMA diagram for the technical texts. (DOCX)

S1 Table Articles quality assessment. (DOCX)

S2 Table Countries list. Distribution of countries classified in Groups A, B, C, D and E by income level, available academic articles and manuals and reported tuberculosis in animals.
(DOCX)
