# Supplementary material for: One Health and surveillance of zoonotic tuberculosis in selected low-income, middle-income and high-income countries: A systematic review
Source: PLoS Negl Trop Dis. 2022 Jun 6;16(6):e0010428. doi: 10.1371/journal.pntd.0010428 (PMC9203019; doi:10.1371/journal.pntd.0010428)
Supplement: S1 Data — (DOCX) [file pntd.0010428.s002.docx]

**Additional file (S1_Data).** Databases and keywords used in the search strategy for systematic review.

| **Databases** | **Keywords** |
| --- | --- |
| Pubmed | (mycobacterium bovis[Title/Abstract]) AND surveillance[Title/Abstract]); (mycobacterium bovis[Title/Abstract]) AND control[Title/Abstract]) AND human[Title/Abstract]; (mycobacterium bovis[Title/Abstract]) AND epidemiology[Title/Abstract]) AND human[Title/Abstract] |
| African Index Medicus | '‘mycobacterium bovis’' |
| Index Medicus for the Eastern Mediterranean Region | tuberculosis [keywords] and bovis [keywords] |
| Index Medicus for the South-East Asian Region | "mycobacterium bovis" |
| Lilacs | (tw:(mycobacterium bovis)) AND (tw:(surveillance)) |
| Embase | ('mycobacterium bovis') AND surveillance:ab,ti; ('mycobacterium bovis') AND control:ab,ti AND human:ab,ti; ('mycobacterium bovis') AND epidemiology:ab,ti AND human:ab,ti |
| Web of Science | (mycobacterium bovis) AND TOPIC: (surveillance) AND TOPIC: (human); (mycobacterium bovis) AND TOPIC: (control) AND TOPIC: (human); (mycobacterium bovis) AND TOPIC: (epidemiology) AND TOPIC: (human) |
| Scielo | (mycobacterium bovis) AND (surveillance); (mycobacterium bovis) AND (control); (mycobacterium bovis) AND (epidemiology) |
