# Supplementary material for: One Health and surveillance of zoonotic tuberculosis in selected low-income, middle-income and high-income countries: A systematic review
Source: PLoS Negl Trop Dis. 2022 Jun 6;16(6):e0010428. doi: 10.1371/journal.pntd.0010428 (PMC9203019; doi:10.1371/journal.pntd.0010428)
Supplement: S1 PRISMA Flow Diagram — (DOCX) [file pntd.0010428.s003.docx]

**Additional file (S1_Prisma) - Flow Diagram – Articles – PRISMA adapted**

Full-text articles excluded, with reasons:

- Clinical case description (n = 19)

- isolation, detection, diagnosis and treatment (n=25)

- genetics, phylogeny and genotyping (n=15)

- pathogenesis and transmission (n=14)

- animal data (n = 10)

- interdisciplinary context with other zoonoses (n=4)

- Other mycobacteria (n = 3)

- multidrug resistance (n =1)

- vaccine and immunology (n=1)

1 from 1 upper middle income country (7.1%)

12 from 9 high income countries (92.9%)

Records identified through database searching:
Index Medicus for the South-East Asian Region (n = 158)

Embase (n=801)

Pubmed (n=561)

Web of Science (n=1,280)

African Index Medicus (n=74)

Index Medicus for the Eastern Mediterranean Region (n=73)

Scielo (n=89)

Lilacs (n=119)

Records after duplicates removed
(n =1,211)

Studies included in qualitative synthesis
(n = 13)

Full-text articles assessed for eligibility
(n = 104)

Records excluded
(n =1,748)

Records screened
(n = 196)

Identification

Eligibility

Included

Screening
