# Supplementary material for: One Health and surveillance of zoonotic tuberculosis in selected low-income, middle-income and high-income countries: A systematic review
Source: PLoS Negl Trop Dis. 2022 Jun 6;16(6):e0010428. doi: 10.1371/journal.pntd.0010428 (PMC9203019; doi:10.1371/journal.pntd.0010428)
Supplement: S2 PRISMA Flow Diagram — (DOCX) [file pntd.0010428.s004.docx]

**Additional file (S2_PRISMA) - Flow Diagram – Technical Texts – PRISMA adapted**

75 countries were not surveyed due language exclusion:

- 27 high income countries (36.0%)

- 22 upper middle income countries (29.3%)

- 21 lower middle income countries (28.0%)

- 5 low income countries (6.7%)

No texts were found in 37 countries:

- 10 high income countries (27.0%)
- 10 upper middle income countries (27.0%)

- 5 lower middle income countries (13.6%)

- 12 low income countries (32.4%)

Final selection: 208 texts from 82 member states
n= 208 (100%)

61 texts from 18 high income countries
n = 61 (29.3%)

59 texts from 23 upper middle income countries
n = 59 (28.4%)

57 texts from 25 lower middle income countries

n = 57 (27.4%)

31 texts from 16 low income countries
n = 31 (14.9%)

Eligibility

Identification and screening

Inclusion criteria:

- Texts written in english, french, italian, portuguese or Spanish

- Texts available at oficial eletronic addressess

Search in the websites of the Ministries of Health of all 194 members states of the World Health Organization

Included
