# Supplementary material for: One Health and surveillance of zoonotic tuberculosis in selected low-income, middle-income and high-income countries: A systematic review
Source: PLoS Negl Trop Dis. 2022 Jun 6;16(6):e0010428. doi: 10.1371/journal.pntd.0010428 (PMC9203019; doi:10.1371/journal.pntd.0010428)
Supplement: S1 Table — (DOCX) [file pntd.0010428.s005.docx]

**Additional file (S1_Table)** Table**:** Quality assessment

| Country | Authors | Year and publication journal | Study Design | Quality assessment tool | Coments |
| --- | --- | --- | --- | --- | --- |
| *High income countries* | | | | | |
| Australia | Cousins D.V. and Dawson D.J. [1] | 1999, International Journal of Tuberculosis and Lung Disease | Cross-sectional study | STROBE check-list | Low risk of bias |
| France | Robert, J. *et al .* [2] | 1999, International Journal of Tuberculous lung Disease | Cross-sectional study | STROBE check-list | Low risk of bias |
| Italy | Lombardi, G. *et al.* [3] | 2017, Epidemiological Infections | Cross-sectional study | STROBE check-list | Low risk of bias |
| Netherlands | Majoor, C.J. *et al.* [4] | 2011, Emerging Infectious Diseases | Cross-sectional study | STROBE check-list | Low risk of bias |
| New Zeland | Baker M.G. *et al.* [5] | 2006, Epidemiology & Infection | Cross-sectional study | STROBE check-list | Low risk of bias |
| Spain | Nebreda-mayoral, T. *et al.* [6] | 2017, Enfermedades Infecciosas y Microbiologia Clínica | Cross-sectional study | STROBE check-list | Low risk of bias |
| United Kingdom | Davidson, J.A. *et al.* [7] | 2017, Emerging Infectious Diseases | Retrospective cohort | STROBE check-list | Low risk of bias |
|  | de la Rua-Domenech, R. *et al* [8]*.* | 2006, Tuberculosis | Review | NA* | NA* |
| United States | Scott, C. *et al.* [9] | 2016, Clinical Infectious Disease | Cross-sectional study | STROBE check-list | Low risk of bias |
|  | Gallivan, M. *et al.* [10] | 2015, Emerging Infectious Diseases | Cross-sectional study | STROBE check-list | Low risk of bias |
|  | Hlavsa, M. C. *et al.* [11] | 2008, Clinical Infectious Disease | Cross-sectional study | STROBE check-list | Low risk of bias |
|  | LoBue, P.A. *et al.* [12] | 2003, International Journal of Tuberculous lung Disease | Cross-sectional study | STROBE check-list | Low risk of bias |
| *High Middle-income countries* | | | | | |
| Mexico | Bobadilla-del Valle, M. *et al*. [13] | 2015, Plos Neglected Tropical Diseases | Cross-sectional study | STROBE check-list | Low risk of bias |

*not applicable.

Reference:

1. Cousins D, Dawson D. Tuberculosis due to *Mycobacterium bovis* in the Australian population: cases recorded during 1970–1994. Int J Tuberc Lung Dis. 1999 Aug;3(8):715-21.
2. Robert J, Boulahbal F, Trystram D, Truffot-Pernot C, de Benoist AC, Vincent V, et al. A national survey of human *Mycobacterium bovis* infection in France. Network of Microbiology Laboratories in France. Int J Tuberc Lung Dis.1999 Aug;3(8):711-4.
3. Lombardi G, Botti I, Pacciarini M, Boniotti M, Roncarati G, Dal Monte P. Five-year surveillance of human tuberculosis caused by *Mycobacterium bovis* in Bologna, Italy: an underestimated problem. Epidemiol Infect. 2017 Oct;145(14):3035-3039.
4. Majoor CJ, Magis-Escurra C, van Ingen J, Boeree MJ, van Soolingen D. Epidemiology of *Mycobacterium bovis* disease in humans, The Netherlands, 1993–2007. Emerg Infect Dis. 2011 Mar;17(3):457-63.
5. Baker MG, Lopez LD, Cannon M, De Lisle G, Collins D. Continuing *Mycobacterium bovis* transmission from animals to humans in New Zealand. Epidemiol Infect.2006 Oct;134(5):1068-73.
6. Nebreda-Mayoral T, Brezmes-Valdivieso MF, Gutiérrez-Zufiaurre N, García-de Cruz S, Labayru-Echeverría C, López-Medrano R, et al. Human *Mycobacterium bovis* infection in Castile and León (Spain), 2006-2015, 2006-2015. Enferm Infecc Microbiol Clin.2019 Jan;37(1):19-24.
7. Davidson JA, Loutet MG, O’Connor C, Kearns C, Smith RMM, Lalor MK, et al. Epidemiology of *Mycobacterium bovis* disease in humans in England, Wales, and Northern Ireland, 2002–2014. Emerg Infect Dis.2017 Mar;23(3):377-386.
8. De la Rua-Domenech R. Human *Mycobacterium bovis* infection in the United Kingdom: incidence, risks, control measures and review of the zoonotic aspects of bovine tuberculosis. Tuberculosis 2006 Mar;86(2):77-109.
9. Scott C, Cavanaugh JS, Pratt R, Silk BJ, LoBue P, Moonan PK. Human tuberculosis caused by *Mycobacterium bovis* in the United States, 2006–2013. Clin Infect Dis. 2016 Sep 1;63(5):594-601.
10. Gallivan M, Shah N, Flood J. Epidemiology of human *Mycobacterium bovis* disease, California, USA, 2003–2011. Emerg Infect Dis. 2015 Mar;21(3):435-43.
11. Hlavsa MC, Moonan PK, Cowan LS, Navin TR, Kammerer JS, Morlock GP, et al. Human tuberculosis due to Mycobacterium bovis in the United States, 1995-2005. Clin Infect Dis.2008 Jul 15;47(2):168-75.
12. LoBue P, Betacourt W, Peter C, Moser K. Epidemiology of Mycobacterium bovis disease in San Diego County, 1994–2000. Int J Tuberc Lung Dis. 2003 Feb;7(2):180-5.
13. Bobadilla-del Valle M, Torres-González P, Cervera-Hernández ME, Martínez-Gamboa A, Crabtree-Ramirez B, Chávez-Mazari B, et al. Trends of *Mycobacterium bovis* isolation and first-line anti-tuberculosis drug susceptibility profile: a fifteen-year laboratory-based surveillance. PLoS Negl Trop Dis2015 Sep 30;9(9):e0004124.
