# Supplementary material for: One Health and surveillance of zoonotic tuberculosis in selected low-income, middle-income and high-income countries: A systematic review
Source: PLoS Negl Trop Dis. 2022 Jun 6;16(6):e0010428. doi: 10.1371/journal.pntd.0010428 (PMC9203019; doi:10.1371/journal.pntd.0010428)
Supplement: S2 Table — Distribution of countries classified in Groups A, B, C, D and E by income level, available academic articles and manuals and reported tuberculosis in animals. (DOCX) [file pntd.0010428.s006.docx]

**(S2_Table):** Distribution of countries classified in Groups A, B, C, D and E by income level, available academic articles and manuals and reported tuberculosis in animals.

| Country | Group | Income* | Academic article available | Manual available | Animal TB data |
| --- | --- | --- | --- | --- | --- |
| Australia | A | High Income | Yes | Yes | Absent |
| Canada | A | High Income | No | Yes | Present |
| France | A | High Income | Yes | Yes | Present |
| Ireland | A | High Income | No | Yes | Present |
| Italy | A | High Income | Yes | Yes | Present |
| Mexico | A | Upper-Middle Income | Yes | Yes | Present |
| Netherlands | A | High Income | Yes | Yes | Absent |
| New Zealand | A | High Income | Yes | Yes | Present |
| Spain | A | High Income | Yes | Yes | Present |
| Switzerland | A | High Income | No | Yes | Absent |
| United Kingdom | A | High Income | Yes | Yes | Present |
| United States | A | High Income | Yes | Yes | Present |
| Algeria | B | Upper-Middle Income | No | Yes | Present |
| Argentina | B | Upper-Middle Income | No | Yes | Present |
| Belgium | B | High Income | No | Yes | Present |
| Botswana | B | Upper-Middle Income | No | Yes | Absent |
| Brazil | B | Upper-Middle Income | No | Yes | Present |
| Burkina Faso | B | Low income | No | Yes | Present |
| Cameroon | B | Lower-Middle Income | No | Yes | Present |
| Colombia | B | Upper-Middle Income | No | Yes | Present |
| Costa Rica | B | Upper-Middle Income | No | Yes | Present |
| Cuba | B | Upper-Middle Income | No | Yes | Absent |
| Ecuador | B | Upper-Middle Income | No | Yes | Present |
| Gambia, The | B | Low income | No | Yes | No information |
| Guatemala | B | Lower-Middle Income | No | Yes | Present |
| India | B | Lower-Middle Income | No | Yes | Present |
| Kenya | B | Lower-Middle Income | No | Yes | Absent |
| Lesotho | B | Lower-Middle Income | No | Yes | Absent |
| Malawi | B | Low income | No | Yes | Present |
| Malta | B | High Income | No | Yes | Present |
| Namibia | B | Upper-Middle Income | No | Yes | Absent |
| Nicaragua | B | Lower-Middle Income | No | Yes | Present |
| Paraguay | B | Upper-Middle Income | No | Yes | Present |
| Portugal | B | High Income | No | Yes | Present |
| Singapore | B | High Income | No | Yes | Present |
| South Africa | B | Upper-Middle Income | No | Yes | Present |
| Sri Lanka | B | Lower-Middle Income | No | Yes | Present |
| Tanzania, United Republic of | B | Low income | No | Yes | Present |
| Tunisia | B | Lower-Middle Income | No | Yes | Present |
| Uganda | B | Low income | No | Yes | Present |
| Uruguay | B | High Income | No | Yes | Present |
| Afghanistan | C | Low income | No | Yes | Present |
| Albania | C | Upper-Middle Income | No | Yes | Present |
| Angola | C | Lower-Middle Income | No | Yes | Present |
| Barbados | C | High Income | No | Yes | Absent |
| Bolivia | C | Lower-Middle Income | No | Yes | Present |
| Burundi | C | Low income | No | Yes | Present |
| Cape Verde | C | Lower-Middle Income | No | Yes | Absent |
| Chile | C | High Income | No | Yes | Present |
| Djibouti | C | Lower-Middle Income | No | Yes | Absent |
| Dominican Republic | C | Upper-Middle Income | No | Yes | Present |
| El Salvador | C | Lower-Middle Income | No | Yes | Present |
| Fiji | C | Upper-Middle Income | No | Yes | Present |
| Gabon | C | Upper-Middle Income | No | Yes | No information |
| Ghana | C | Lower-Middle Income | No | Yes | Present |
| Grenada | C | Upper-Middle Income | No | Yes | No information |
| Guinea | C | Low income | No | Yes | No information |
| Guinea-Bissau | C | Low income | No | Yes | Present |
| Haiti | C | Low income | No | Yes | Present |
| Honduras | C | Lower-Middle Income | No | Yes | Present |
| Liberia | C | Low income | No | Yes | Present |
| Mali | C | Low income | No | Yes | No information |
| Mauritius | C | Upper-Middle Income | No | Yes | Absent |
| Mozambique | C | Low income | No | Yes | Present |
| Nauru | C | Upper-Middle Income | No | Yes | No information |
| Nigeria | C | Lower-Middle Income | No | Yes | Present |
| Niue | C | Lower-Middle Income | No | Yes | No information |
| Pakistan | C | Lower-Middle Income | No | Yes | Present |
| Papua New Guinea | C | Lower-Middle Income | No | Yes | Absent |
| Peru | C | Upper-Middle Income | No | Yes | Present |
| Philippines | C | Lower-Middle Income | No | Yes | Present |
| Rwanda | C | Low income | No | Yes | Present |
| Samoa | C | Upper-Middle Income | No | Yes | Absent |
| Sao Tome and Principe | C | Lower-Middle Income | No | Yes | No information |
| Senegal | C | Low income | No | Yes | Present |
| South Sudan | C | Lower-Middle Income | No | Yes | No information |
| St. Lucia | C | Upper-Middle Income | No | Yes | No information |
| Sudan | C | Lower-Middle Income | No | Yes | Absent |
| Swaziland | C | Lower-Middle Income | No | Yes | Present |
| Venezuela | C | Upper-Middle Income | No | Yes | Present |
| Zambia | C | Lower-Middle Income | No | Yes | Present |
| Zimbabwe | C | Low income | No | Yes | Absent |
| Benin | D | Low income | No | No | Present |
| Central African Republic | D | Low income | No | No | Present |
| Chad | D | Low income | No | No | Present |
| Comoros | D | Low income | No | No | No information |
| Congo (Democratic Republic of the) | D | Low income | No | No | Present |
| Congo (Republic of the) | D | Lower-Middle Income | No | No | Present |
| Cote d'Ivoire | D | Lower-Middle Income | No | No | Present |
| Madagascar | D | Low income | No | No | Present |
| Niger | D | Low income | No | No | Present |
| Panama | D | Upper-Middle Income | No | No | Present |
| Sierra Leone | D | Low income | No | No | Absent |
| Togo | D | Low income | No | No | Present |
| Andorra | E | High Income | No | No | Absent |
| Antigua and Barbuda | E | High Income | No | No | No information |
| Bahamas, The | E | High Income | No | No | Absent |
| Belize | E | Upper-Middle Income | No | No | Present |
| Cook Islands | E | Upper-Middle Income | No | No | Absent |
| Dominica | E | Upper-Middle Income | No | No | No information |
| East Timor | E | Lower-Middle Income | No | No | No information |
| Equatorial Guinea | E | Upper-Middle Income | No | No | No information |
| Federated States of Micronesia | E | Lower-Middle Income | No | No | Absent |
| Guyana | E | Upper-Middle Income | No | No | Present |
| Jamaica | E | Upper-Middle Income | No | No | Absent |
| Kiribati | E | Lower-Middle Income | No | No | Absent |
| Luxembourg | E | High Income | No | No | No information |
| Marshall Islands | E | Upper-Middle Income | No | No | Absent |
| Monaco | E | High Income | No | No | No information |
| Palau | E | High Income | No | No | Absent |
| San Marino | E | High Income | No | No | Absent |
| Seychelles | E | High Income | No | No | No information |
| Solomon Islands | E | Lower-Middle Income | No | No | No information |
| St. Kitts and Nevis | E | High Income | No | No | No information |
| St. Vincent and the Grenadines | E | Upper-Middle Income | No | No | No information |
| Tonga | E | Upper-Middle Income | No | No | Absent |
| Trinidad and Tobago | E | High Income | No | No | Absent |
| Tuvalu | E | Upper-Middle Income | No | No | No information |
| Vanuatu | E | Lower-Middle Income | No | No | Absent |

* Income classification according to the World Bank.

** Source: articles and technical texts included in the study.
